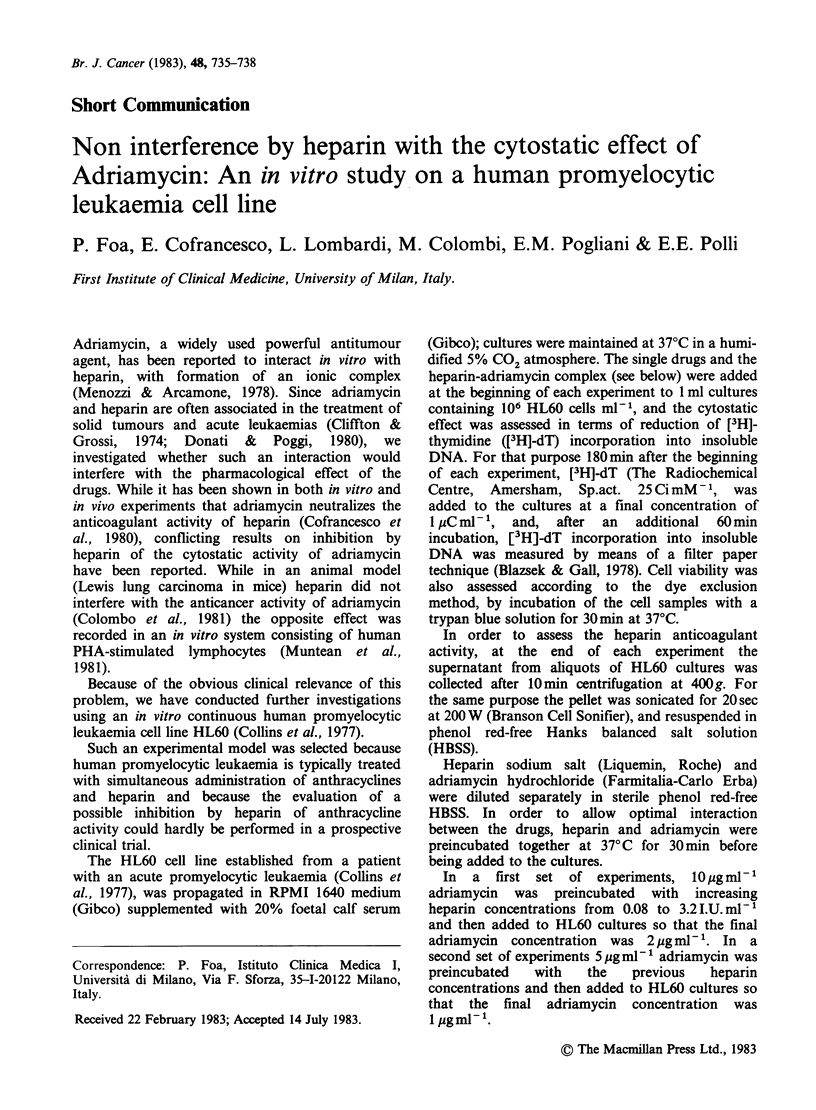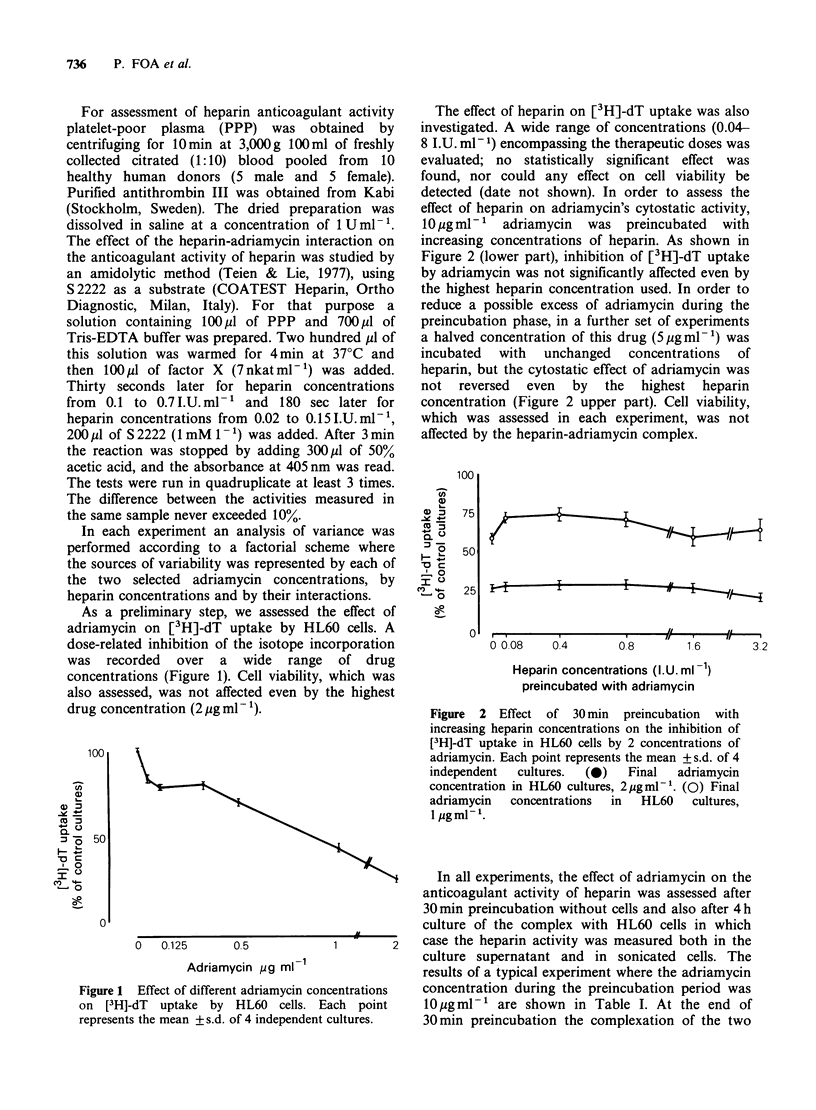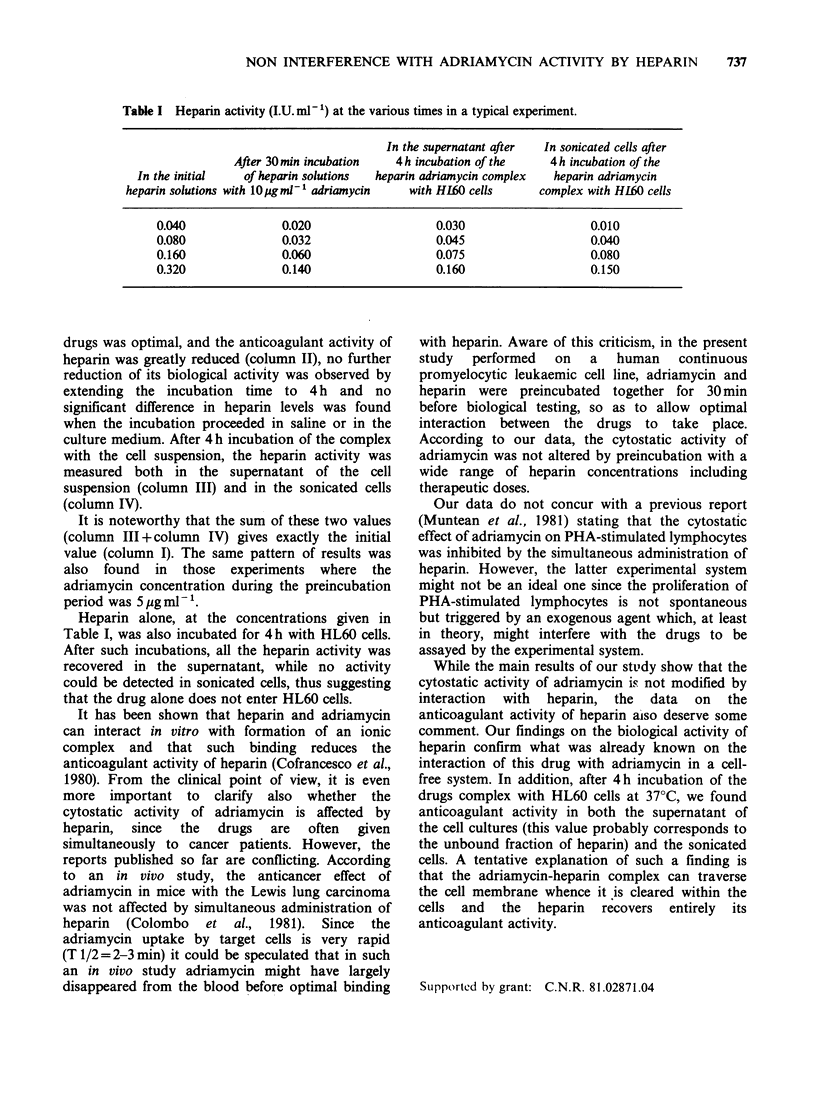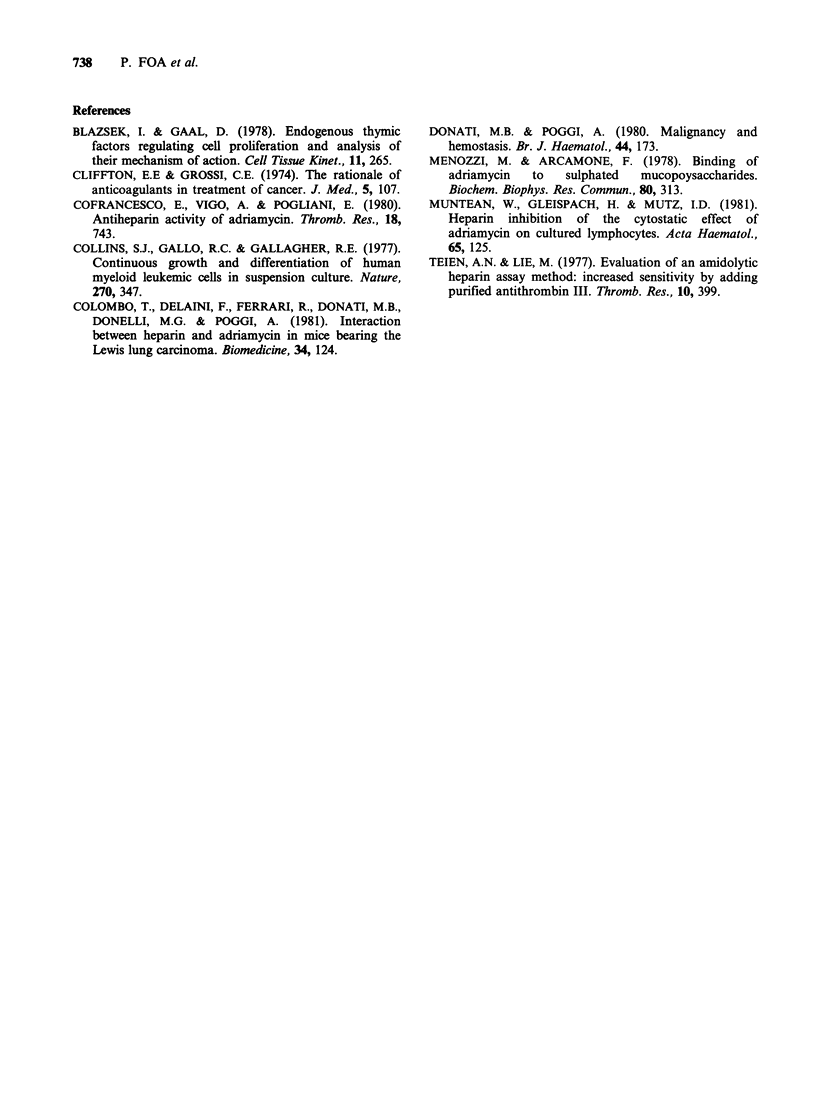# Non interference by heparin with the cytostatic effect of adriamycin: an in vitro study on a human promyelocytic leukaemia cell line.

**DOI:** 10.1038/bjc.1983.258

**Published:** 1983-11

**Authors:** P. Foa, E. Cofrancesco, L. Lombardi, M. Colombi, E. M. Pogliani, E. E. Polli


*Br. J. Cancer* (1983), **48**, 735–738

**Short Communication**

### Non interference by heparin with the cytostatic effect of Adriamycin: An *in vitro* study on a human promyelocytic leukaemia cell line

P. Foa, E. Cofrancesco, L. Lombardi, M. Colombi, E.M. Pogliani & E.E. Polli

*First Institute of Clinical Medicine, University of Milan, Italy.*

Adriamycin, a widely used powerful antitumour agent, has been reported to interact *in vitro* with heparin, with formation of an ionic complex (Menozzi & Arcamone, 1978). Since adriamycin and heparin are often associated in the treatment of solid tumours and acute leukaemias (Cliffton & Grossi, 1974; Donati & Poggi, 1980), we investigated whether such an interaction would interfere with the pharmacological effect of the drugs. While it has been shown in both *in vitro* and *in vivo* experiments that adriamycin neutralizes the anticoagulant activity of heparin (Cofrancesco *et al.*, 1980), conflicting results on inhibition by heparin of the cytostatic activity of adriamycin have been reported. While in an animal model (Lewis lung carcinoma in mice) heparin did not interfere with the anticancer activity of adriamycin (Colombo *et al.*, 1981) the opposite effect was recorded in an *in vitro* system consisting of human PHA-stimulated lymphocytes (Muntean *et al.*, 1981).

Because of the obvious clinical relevance of this problem, we have conducted further investigations using an *in vitro* continuous human promyelocytic leukaemia cell line HL60 (Collins *et al.*, 1977).

Such an experimental model was selected because human promyelocytic leukaemia is typically treated with simultaneous administration of anthracyclines and heparin and because the evaluation of a possible inhibition by heparin of anthracycline activity could hardly be performed in a prospective clinical trial.

The HL60 cell line established from a patient with an acute promyelocytic leukaemia (Collins *et al.*, 1977), was propagated in RPMI 1640 medium (Gibco) supplemented with 20% foetal calf serum (Gibco); cultures were maintained at 37°C in a humidified 5% $CO_2$ atmosphere. The single drugs and the heparin-adriamycin complex (see below) were added at the beginning of each experiment to 1 ml cultures containing $10^6$ HL60 cells $ml^{-1}$, and the cytostatic effect was assessed in terms of reduction of [$^3$H]-thymidine ([$^3$H]-dT) incorporation into insoluble DNA. For that purpose 180 min after the beginning of each experiment, [$^3$H]-dT (The Radiochemical Centre, Amersham, Sp.act. 25 Ci mM$^{-1}$, was added to the cultures at a final concentration of 1 $\mu$C ml$^{-1}$, and, after an additional 60 min incubation, [$^3$H]-dT incorporation into insoluble DNA was measured by means of a filter paper technique (Blazsek & Gall, 1978). Cell viability was also assessed according to the dye exclusion method, by incubation of the cell samples with a trypan blue solution for 30 min at 37°C.

In order to assess the heparin anticoagulant activity, at the end of each experiment the supernatant from aliquots of HL60 cultures was collected after 10 min centrifugation at 400 *g*. For the same purpose the pellet was sonicated for 20 sec at 200 W (Branson Cell Sonifier), and resuspended in phenol red-free Hanks balanced salt solution (HBSS).

Heparin sodium salt (Liquemin, Roche) and adriamycin hydrochloride (Farmitalia-Carlo Erba) were diluted separately in sterile phenol red-free HBSS. In order to allow optimal interaction between the drugs, heparin and adriamycin were preincubated together at 37°C for 30 min before being added to the cultures.

In a first set of experiments, 10 $\mu$g ml$^{-1}$ adriamycin was preincubated with increasing heparin concentrations from 0.08 to 3.2 I.U. ml$^{-1}$ and then added to HL60 cultures so that the final adriamycin concentration was 2 $\mu$g ml$^{-1}$. In a second set of experiments 5 $\mu$g ml$^{-1}$ adriamycin was preincubated with the previous heparin concentrations and then added to HL60 cultures so that the final adriamycin concentration was 1 $\mu$g ml$^{-1}$.

Correspondence: P. Foa, Istituto Clinica Medica I, Università di Milano, Via F. Sforza, 35–I-20122 Milano, Italy.

Received 22 February 1983; Accepted 14 July 1983.

© The Macmillan Press Ltd., 1983

For assessment of heparin anticoagulant activity platelet-poor plasma (PPP) was obtained by centrifuging for 10 min at 3,000 g 100 ml of freshly collected citrated (1:10) blood pooled from 10 healthy human donors (5 male and 5 female). Purified antithrombin III was obtained from Kabi (Stockholm, Sweden). The dried preparation was dissolved in saline at a concentration of $1\,U\,ml^{-1}$. The effect of the heparin-adriamycin interaction on the anticoagulant activity of heparin was studied by an amidolytic method (Teien & Lie, 1977), using S 2222 as a substrate (COATEST Heparin, Ortho Diagnostic, Milan, Italy). For that purpose a solution containing 100 $\mu$l of PPP and 700 $\mu$l of Tris-EDTA buffer was prepared. Two hundred $\mu$l of this solution was warmed for 4 min at 37°C and then 100 $\mu$l of factor X ($7\,nkat\,ml^{-1}$) was added. Thirty seconds later for heparin concentrations from 0.1 to $0.7\,I.U.\,ml^{-1}$ and 180 sec later for heparin concentrations from 0.02 to $0.15\,I.U.\,ml^{-1}$, 200 $\mu$l of S 2222 ($1\,mM\,l^{-1}$) was added. After 3 min the reaction was stopped by adding 300 $\mu$l of 50% acetic acid, and the absorbance at 405 nm was read. The tests were run in quadruplicate at least 3 times. The difference between the activities measured in the same sample never exceeded 10%.

In each experiment an analysis of variance was performed according to a factorial scheme where the sources of variability was represented by each of the two selected adriamycin concentrations, by heparin concentrations and by their interactions.

As a preliminary step, we assessed the effect of adriamycin on [$^3$H]-dT uptake by HL60 cells. A dose-related inhibition of the isotope incorporation was recorded over a wide range of drug concentrations (Figure 1). Cell viability, which was also assessed, was not affected even by the highest drug concentration ($2\,\mu g\,ml^{-1}$).

**Figure 1** Effect of different adriamycin concentrations on [$^3$H]-dT uptake by HL60 cells. Each point represents the mean ±s.d. of 4 independent cultures.

The effect of heparin on [$^3$H]-dT uptake was also investigated. A wide range of concentrations (0.04–8 $I.U.\,ml^{-1}$) encompassing the therapeutic doses was evaluated; no statistically significant effect was found, nor could any effect on cell viability be detected (date not shown). In order to assess the effect of heparin on adriamycin's cytostatic activity, $10\,\mu g\,ml^{-1}$ adriamycin was preincubated with increasing concentrations of heparin. As shown in Figure 2 (lower part), inhibition of [$^3$H]-dT uptake by adriamycin was not significantly affected even by the highest heparin concentration used. In order to reduce a possible excess of adriamycin during the preincubation phase, in a further set of experiments a halved concentration of this drug ($5\,\mu g\,ml^{-1}$) was incubated with unchanged concentrations of heparin, but the cytostatic effect of adriamycin was not reversed even by the highest heparin concentration (Figure 2 upper part). Cell viability, which was assessed in each experiment, was not affected by the heparin-adriamycin complex.

**Figure 2** Effect of 30 min preincubation with increasing heparin concentrations on the inhibition of [$^3$H]-dT uptake in HL60 cells by 2 concentrations of adriamycin. Each point represents the mean ±s.d. of 4 independent cultures. (●) Final adriamycin concentration in HL60 cultures, $2\,\mu g\,ml^{-1}$. (○) Final adriamycin concentrations in HL60 cultures, $1\,\mu g\,ml^{-1}$.

In all experiments, the effect of adriamycin on the anticoagulant activity of heparin was assessed after 30 min preincubation without cells and also after 4 h culture of the complex with HL60 cells in which case the heparin activity was measured both in the culture supernatant and in sonicated cells. The results of a typical experiment where the adriamycin concentration during the preincubation period was $10\,\mu g\,ml^{-1}$ are shown in Table I. At the end of 30 min preincubation the complexation of the two

**Table I** Heparin activity (I.U. $ml^{-1}$) at the various times in a typical experiment.

| *In the initial heparin solutions* | *After 30 min incubation of heparin solutions with 10 μg ml⁻¹ adriamycin* | *In the supernatant after 4 h incubation of the heparin adriamycin complex with HL60 cells* | *In sonicated cells after 4 h incubation of the heparin adriamycin complex with HL60 cells* |
|---|---|---|---|
| 0.040 | 0.020 | 0.030 | 0.010 |
| 0.080 | 0.032 | 0.045 | 0.040 |
| 0.160 | 0.060 | 0.075 | 0.080 |
| 0.320 | 0.140 | 0.160 | 0.150 |

drugs was optimal, and the anticoagulant activity of heparin was greatly reduced (column II), no further reduction of its biological activity was observed by extending the incubation time to 4 h and no significant difference in heparin levels was found when the incubation proceeded in saline or in the culture medium. After 4 h incubation of the complex with the cell suspension, the heparin activity was measured both in the supernatant of the cell suspension (column III) and in the sonicated cells (column IV).

It is noteworthy that the sum of these two values (column III + column IV) gives exactly the initial value (column I). The same pattern of results was also found in those experiments where the adriamycin concentration during the preincubation period was 5 $\mu g\,ml^{-1}$.

Heparin alone, at the concentrations given in Table I, was also incubated for 4 h with HL60 cells. After such incubations, all the heparin activity was recovered in the supernatant, while no activity could be detected in sonicated cells, thus suggesting that the drug alone does not enter HL60 cells.

It has been shown that heparin and adriamycin can interact *in vitro* with formation of an ionic complex and that such binding reduces the anticoagulant activity of heparin (Cofrancesco *et al.*, 1980). From the clinical point of view, it is even more important to clarify also whether the cytostatic activity of adriamycin is affected by heparin, since the drugs are often given simultaneously to cancer patients. However, the reports published so far are conflicting. According to an *in vivo* study, the anticancer effect of adriamycin in mice with the Lewis lung carcinoma was not affected by simultaneous administration of heparin (Colombo *et al.*, 1981). Since the adriamycin uptake by target cells is very rapid (T 1/2 = 2–3 min) it could be speculated that in such an *in vivo* study adriamycin might have largely disappeared from the blood before optimal binding with heparin. Aware of this criticism, in the present study performed on a human continuous promyelocytic leukaemic cell line, adriamycin and heparin were preincubated together for 30 min before biological testing, so as to allow optimal interaction between the drugs to take place. According to our data, the cytostatic activity of adriamycin was not altered by preincubation with a wide range of heparin concentrations including therapeutic doses.

Our data do not concur with a previous report (Muntean *et al.*, 1981) stating that the cytostatic effect of adriamycin on PHA-stimulated lymphocytes was inhibited by the simultaneous administration of heparin. However, the latter experimental system might not be an ideal one since the proliferation of PHA-stimulated lymphocytes is not spontaneous but triggered by an exogenous agent which, at least in theory, might interfere with the drugs to be assayed by the experimental system.

While the main results of our study show that the cytostatic activity of adriamycin is not modified by interaction with heparin, the data on the anticoagulant activity of heparin also deserve some comment. Our findings on the biological activity of heparin confirm what was already known on the interaction of this drug with adriamycin in a cell-free system. In addition, after 4 h incubation of the drugs complex with HL60 cells at 37°C, we found anticoagulant activity in both the supernatant of the cell cultures (this value probably corresponds to the unbound fraction of heparin) and the sonicated cells. A tentative explanation of such a finding is that the adriamycin-heparin complex can traverse the cell membrane whence it is cleared within the cells and the heparin recovers entirely its anticoagulant activity.

Supported by grant: C.N.R. 81.02871.04

### References

BLAZSEK, I. & GAAL, D. (1978). Endogenous thymic factors regulating cell proliferation and analysis of their mechanism of action. *Cell Tissue Kinet.*, **11**, 265.

CLIFFTON, E.E & GROSSI, C.E. (1974). The rationale of anticoagulants in treatment of cancer. *J. Med.*, **5**, 107.

COFRANCESCO, E., VIGO, A. & POGLIANI, E. (1980). Antiheparin activity of adriamycin. *Thromb. Res.*, **18**, 743.

COLLINS, S.J., GALLO, R.C. & GALLAGHER, R.E. (1977). Continuous growth and differentiation of human myeloid leukemic cells in suspension culture. *Nature*, **270**, 347.

COLOMBO, T., DELAINI, F., FERRARI, R., DONATI, M.B., DONELLI, M.G. & POGGI, A. (1981). Interaction between heparin and adriamycin in mice bearing the Lewis lung carcinoma. *Biomedicine*, **34**, 124.

DONATI, M.B. & POGGI, A. (1980. Malignancy and hemostasis. *Br. J. Haematol.*, **44**, 173.

MENOZZI, M. & ARCAMONE, F. (1978). Binding of adriamycin to sulphated mucopoysaccharides. *Biochem. Biophys. Res. Commun.*, **80**, 313.

MUNTEAN, W., GLEISPACH, H. & MUTZ, I.D. (1981). Heparin inhibition of the cytostatic effect of adriamycin on cultured lymphocytes. *Acta Haematol.*, **65**, 125.

TEIEN, A.N. & LIE, M. (1977). Evaluation of an amidolytic heparin assay method: increased sensitivity by adding purified antithrombin III. *Thromb. Res.*, **10**, 399.